# Reply to: “Correlation between paddy rice growth and satellite-observed methane column abundance does not imply causation”

**DOI:** 10.1038/s41467-021-21437-4

**Published:** 2021-02-19

**Authors:** Geli Zhang, Xiangming Xiao, Jinwei Dong, Yao Zhang, Fengfei Xin, Yuanwei Qin, Russell B. Doughty, Berrien Moore

**Affiliations:** 1 College of Land Science and Technology, China Agricultural University, Beijing, China; 2 Department of Microbiology and Plant Biology, and Center for Spatial Analysis, University of Oklahoma, Norman, OK USA; 3 Key Laboratory of Land Surface Pattern and Simulation, Institute of Geographic Sciences and Natural Resources Research, Chinese Academy of Sciences, Beijing, China; 4 Climate and Ecosystem Sciences Division, Lawrence Berkeley National Laboratory, Berkeley, CA USA; 5 Ministry of Education Key Laboratory of Biodiversity Science and Ecological Engineering, Institute of Biodiversity Science, Fudan University, Shanghai, China; 6 Division of Geological and Planetary Sciences, California Institute of Technology, Pasadena, CA USA; 7 College of Atmospheric and Geographic Sciences, University of Oklahoma, Norman, OK USA

**Keywords:** Carbon cycle, Carbon cycle, Agriculture

**Replying** **to** Z. Zeng et al. *Nature Communications* 10.1038/s41467-021-21434-7 (2021)

Atmospheric methane concentration (XCH_4_) measured by satellite-based sensors is affected by in situ CH_4_ emissions (local fluxes), atmospheric chemistry, and atmospheric transport (external fluxes). Based on annual paddy rice maps at the 500-m spatial resolution, our study^1^ investigated the spatial and seasonal consistency between rice paddies and atmospheric methane concentration in monsoon Asia. In our study^1^, we implied that annual paddy rice maps at moderate spatial resolution (500 m) may be used to increase the accuracy of and reduce the uncertainty in modeling XCH_4_ dynamics over those areas with moderate to large proportions of rice paddy. We appreciate the comments from Zeng et al.^2^ as their work used the Greenhouse Gas Framework – Flux (GHGF-Flux) forward model, a state-of-the-art flux inversion system used by the National Aeronautics and Space Administration (NASA) Carbon Monitoring System program. Their results, analyses, and discussion offer insights into how the GHGF-Flux model assesses the relative roles of in situ CH_4_ emissions, atmospheric chemistry, and atmospheric transport in the spatial-temporal dynamics of XCH_4_. Here, we provide our responses to the two concerns raised by Zeng et al.^2^, which may further unveil the role of paddy rice agriculture in the seasonal dynamics and spatial distributions of XCH_4_ in monsoon Asia.

Zeng et al.^2^ analyzed the relative contributions of locally emitted CH_4_ fluxes and externally transported CH_4_ fluxes to the seasonal cycle of XCH_4_ in the four regions of interest (ROIs): Northeast China, Southeast China, Northwest India, and North Bangladesh. They reported that externally transported CH_4_ fluxes contributed more to the seasonal cycle of XCH_4_ than did locally emitted CH_4_ fluxes in Northeast China, Southeast China, and Northwest India, but the relative roles of these two CH_4_ fluxes were comparable in North Bangladesh^2^. Our study reported that the seasonal dynamics of XCH_4_ and paddy rice growth were consistent across the 0.5° gridcells with moderate to high proportions of rice paddy (area percentage >10% within gridcells)^1^. This discrepancy in the role of rice paddies in seasonal dynamics of XCH_4_ between Zeng et al.^2^ and our study^1^ can be attributed to three factors.

First, the area of the ROIs used in Zeng et al.^2^ (Fig. 1a) was substantially larger than that used in our study^1^. Larger ROIs have much lower proportions of rice paddy area (Fig. 1g–n). Statistically, average values over very large ROIs would dampen localized seasonal variations, which often leads to failure to identify hot spots within ROIs^3^. Second, the spatial resolution of the gridded data we used in our study^1^ was finer than that used by Zeng et al.^2^. The GHGF-Flux CH_4_ inversion used by Zeng et al.^2^ was carried out at 2° × 2.5° horizontal spatial resolution, which is much coarser than the spatial resolution of the XCH_4_ data from the SCIAMACHY sensors (0.5° × 0.5°) that comprised our ROIs^1^. Given that there are many land cover types in monsoon Asia, larger gridcells would have lower proportions of rice paddy area (Fig. 1g–n), and would thus diminish the local contribution of CH_4_ emission from rice paddy on the seasonal cycle of XCH_4_. As shown in Fig. 1a by Zeng et al.^2^, the relative contribution of locally emitted CH_4_ fluxes to the seasonal cycle of XCH_4_ increased with the proportion of rice paddy area within the ROIs. The North Bangladesh ROI is a good example. Rice paddy in Bangladesh accounts for about 68% of the country’s land area (Fig. 1e), and it occupies a large proportion (~22%) of the 2° × 2.5° gridcells in the ROI (Fig. 1m) compared to the gridcells in the other ROIs (Fig. 1k, l, n). Thus, the comparable relative contributions of local and external CH_4_ fluxes to the seasonal cycle of XCH_4_ in the North Bangladesh ROI (see Fig. 1a in Zeng et al.^2^) are likely driven in part by the region’s high rice paddy proportion. Third, the GHGF-Flux model used the CarbonTracker-CH_4_ emission from EDGAR 3.2FT2000 as prior CH_4_ emission estimates of rice paddy, enteric fermentation, and animal waste. The EDGAR dataset’s estimates of CH_4_ emissions from rice paddy are based on paddy rice area from agricultural statistics at various administrative levels^4,5^, which often cannot resolve the spatial distribution of paddy rice area at a 0.5° spatial resolution. The spatial heterogeneity of CH_4_ emission sources cannot be captured using larger ROIs, coarser gridcells, and inaccurate model inputs. In addition, XCH_4_ is theoretically calculated as the total CH_4_ across different altitudes. However, the SCIAMACHY XCH_4_ retrieval is mainly based on the short-wavelength infrared band (SWIR), which is more indicative of CH_4_ at lower altitudes down to the surface^6^.

**Fig. 1:**
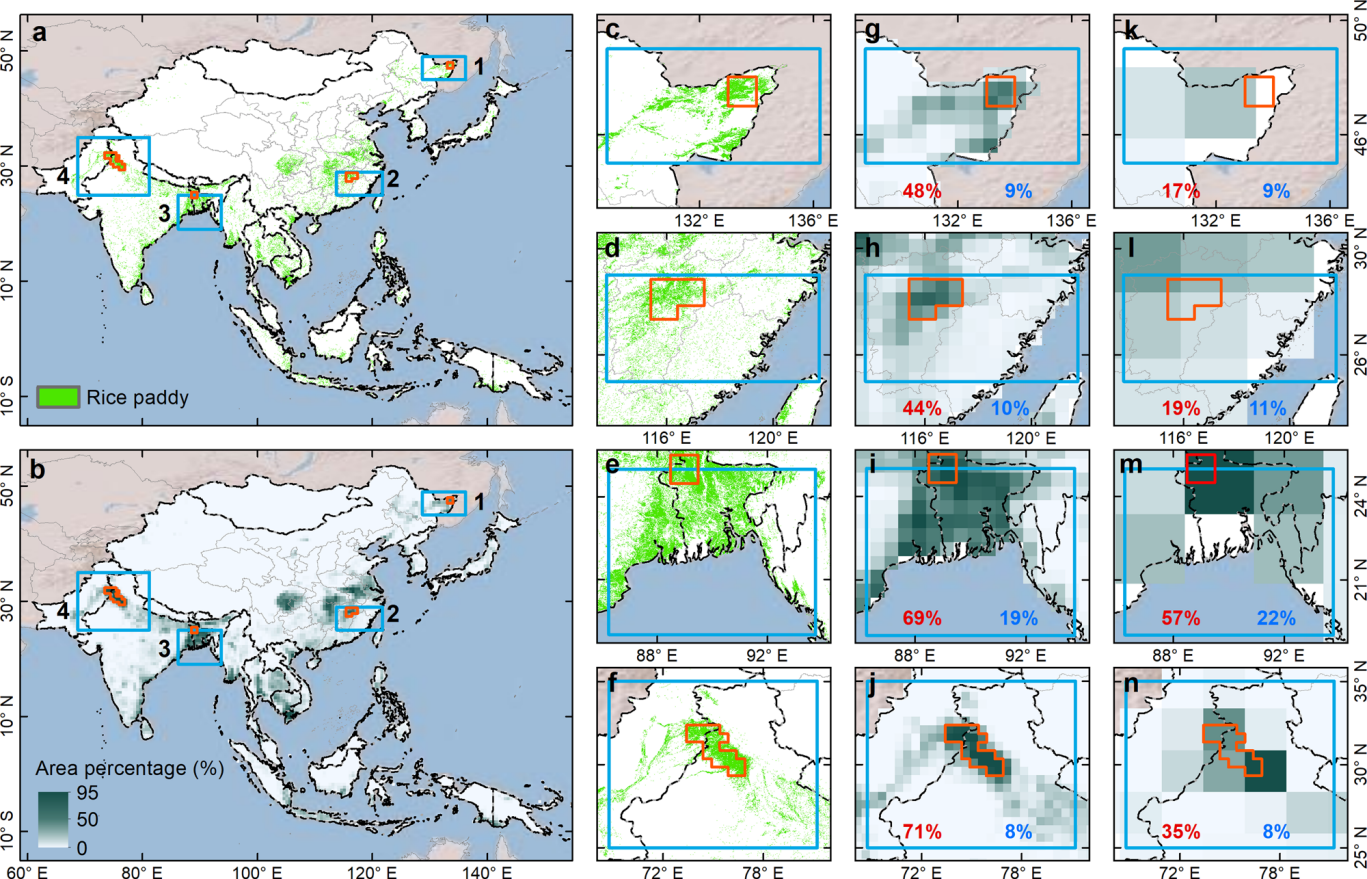
Potential effects of different sizes of regions of interest (ROIs) with varied footprints of rice paddies. The red and blue ROIs are from our study^1^ and Zeng et al.^2^, respectively. The paddy rice maps were retrieved from MODIS data with the 500-m spatial resolution (**a**) and 0.5° spatial resolution (**b**) in monsoon Asia in 2010, respectively. Detailed spatial distributions of rice paddies in local regions labeled with blue rectangles and spatial resolutions of 500 m (**c**–**f**), 0.5° (**g**–**j**), and 2° × 2.5° (**k**–**n**). The red and blue numbers show the mean of rice paddy area proportion in ROIs with red and blue color, respectively.

Zeng et al.^2^ further analyzed the seasonal dynamics of XCH_4_ from the four ROIs and four latitudinal zones (10° interval) that were centered on the four ROIs during 2003–2011 (see Fig. 1b by Zeng et al.^2^), and claimed that there were strong agreements between the ROIs and latitudinal zones. Unfortunately, the authors failed to recognize that the Southeast China ROI had very different seasonal dynamics between the ROI (two XCH_4_ peaks in one year) and latitudinal zonal XCH_4_ (one XCH_4_ peak in one year) (Fig. 1b by Zeng et al.^2^ and Fig. 2 here). The timing of the two XCH_4_ peaks in one year is actually related to the double paddy rice cropping system in South China (Supplementary Fig. 1), which we explained in our study^1^. This noticeable two-peak seasonal dynamic in the Southeast China ROI further highlights the importance of annual paddy rice maps at moderate spatial resolution (500 m) in understanding the seasonal dynamics of paddy rice CH_4_ emissions and XCH_4_.

**Fig. 2:**
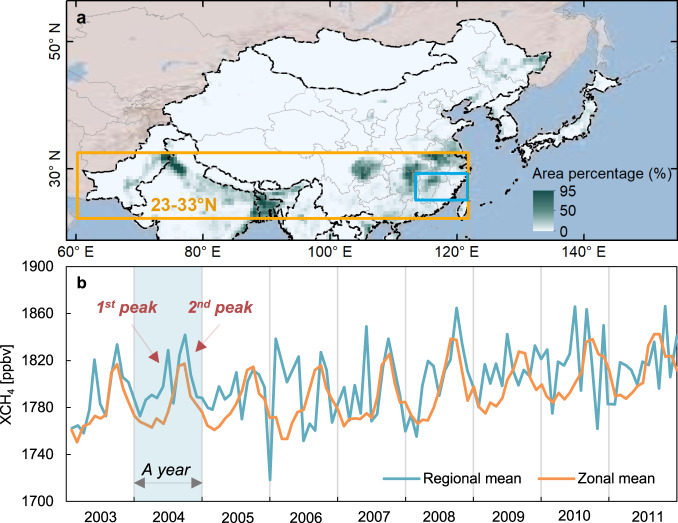
Monthly averaged XCH_4_ in the region of interest (ROI) of Southeast China and the latitudinal zone centered on the ROI according to Zeng et al.^2^. **a** The MODIS-based paddy rice map at 0.5° resolution in monsoon Asia in 2010. The orange rectangle shows the latitudinal zone of 23°–33°N centered on the Southeast China ROI. The blue rectangle shows the boundary of the Southeast China ROI from Zeng et al.^2^. **b** Monthly averaged XCH_4_ in the ROI and the latitudinal zone according to Zeng et al.^2^. The regional mean in **b** is the averaged value of XCH_4_ in ROI, and the zonal mean in **b** is the averaged XCH_4_ over the latitudinal zone centered on the ROI, which is from Zeng et al.’s paper^2^.

Our study reported that there were consistent spatial distributions between XCH_4_ and paddy rice area across those 0.5° gridcells with relatively moderate to high proportions of rice paddy (area percentage >10% within gridcells)^1^. Zeng et al.^2^ analyzed the spatial distributions of XCH_4_ and EDGAR-based CH_4_ emissions from agricultural and non-agriculture sectors for all 1° gridcells in monsoon Asia in 2010 and reported that the spatial distribution of XCH_4_ correlated with CH_4_ emissions from both agricultural and non-agricultural sectors. We recognize that paddy rice area is one of many factors that affect the spatial distribution of XCH_4_ in dense rice paddy regions; however, EDGAR’s use of agricultural statistical data at administrative levels (e.g., national, state or province)^4,5^ precludes accurate resolution of the geographic (or spatial) distribution of different CH_4_ emission sources. Furthermore, the 1° gridcell analyses of the EDGAR data in Zeng et al.^2^ cannot reflect the spatial heterogeneity of CH_4_ emissions from different sources within the gridcells. Thus, the higher consistency between non-agricultural CH_4_ emissions and XCH_4_ reported in Zeng et al.^2^ does not refute our finding on the role of CH_4_ emission from rice paddies. The finer spatial resolution data of CH_4_ emissions from rice paddies could rather improve the EDGAR data, and thus improve our understanding of the relative role of agricultural and non-agricultural CH_4_ emissions in the spatial distribution of XCH_4_.

In summary, we recognize the importance of the GHGF-Flux model for CH_4_ flux inversion, atmospheric chemistry, atmospheric transport, and attribution of CH_4_ emissions to various sources. Together, the results from Zeng et al.^2^ using the GHGF-Flux model and our study^1^ based on higher resolution paddy rice maps and satellite observations highlight the importance of the high-resolution paddy rice maps to understanding the spatial distribution and seasonal dynamics of XCH_4_. Annual paddy rice maps at moderate and high spatial resolutions can be used to further improve CH_4_ emission estimates from rice paddies in the EDGAR dataset and to better understand the relationships between the spatial distribution and seasonal dynamics of XCH_4_ from the TROPOspheric Monitoring Instrument (TROPOMI, 7 × 7 km spatial resolution) and rice paddies in monsoon Asia.

## Supplementary information

Supplementary Information

## Data Availability

The paddy rice maps can be accessed by contacting Geli Zhang, Xiangming Xiao, or Jinwei Dong. All the relevant data from this study are also available from the corresponding authors upon request.

## Code availability

The code used in this study can be obtained by contacting the corresponding authors.
