# Supplementary material for: Reply to: “Correlation between paddy rice growth and satellite-observed methane column abundance does not imply causation”
Source: Nat Commun. 2021 Feb 19;12:1189. doi: 10.1038/s41467-021-21437-4 (PMC7896092; doi:10.1038/s41467-021-21437-4)
Supplement: Supplementary file 1 — Supplementary Information [file 41467_2021_21437_MOESM1_ESM.pdf]

Supporting Information

Reply to "Correlation is not causation: Caution in correlating paddy rice growth with satellite-observed methane column abundance"

Zhang, et al.

Includes:

Supplementary Figure 1

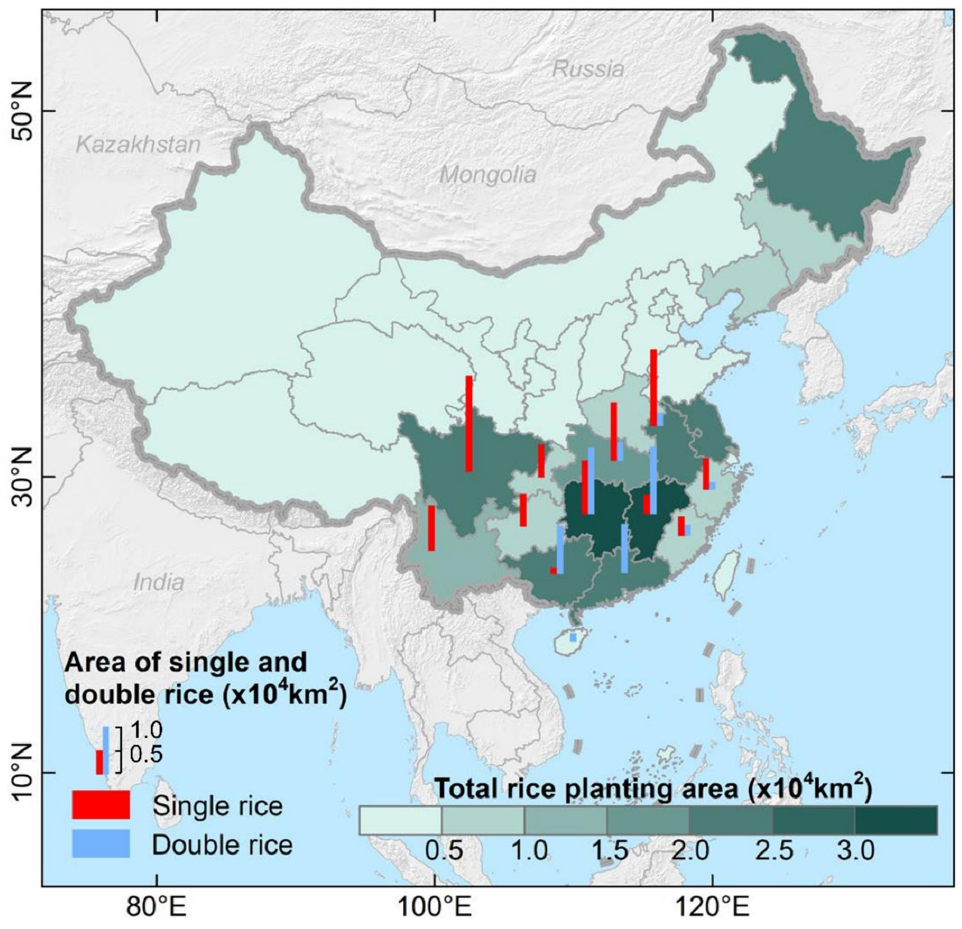

Supplementary Figure 1. Spatial distribution of multi-year averaged paddy rice planting area and rice cropping systems at the provincial scale during 2003 – 2011 in China. Paddy rice planting area and cropping intensity (single- and double-cropping) data were derived from the Statistical Yearbook of China. Note that the EDGAR dataset used province-level agricultural statistical data of paddy rice for China[1,2].

Supplementary References:

1 Peng, S. S. *et al.* Inventory of anthropogenic methane emissions in mainland China from 1980 to 2010. *Atmospheric Chemistry and Physics* **16**, 14545-14562, doi:10.5194/acp-16-14545-2016 (2016).

2 Janssens-Maenhout, G. *et al.* EDGAR v4.3.2 Global Atlas of the three major greenhouse gas emissions for the period 1970-2012. *Earth System Science Data* **11**, 959-1002, doi:10.5194/essd-11-959-2019 (2019).
